# Differentiation of Intracranial Dural Metastases and Meningiomas Using DSC Perfusion MRI and Machine Learning

**DOI:** 10.3390/diagnostics16050781

**Published:** 2026-03-05

**Authors:** Seyit Erol, Halil Özer, Ahmet Baytok, Ayşe Arı, Hakan Cebeci

**Affiliations:** Department of Radiology, Faculty of Medicine, Selcuk University, Konya 42130, Türkiye

**Keywords:** dural metastases, meningioma, DSC perfusion MRI, machine learning

## Abstract

**Background/Objectives:** To assess the diagnostic performance of dynamic susceptibility contrast (DSC) perfusion MRI parameters and machine learning methods for differentiating intracranial dural metastases (IDMs) from meningiomas. **Methods:** This retrospective diagnostic accuracy study included 56 patients (mean age: 57.6 ± 11.2 years; 20 men) with dural-based intracranial lesions (65 lesions): 18 patients with IDM (27 lesions) and 38 patients with meningiomas (38 lesions). All patients underwent DSC perfusion MRI. Relative cerebral blood volume (rCBV), relative cerebral blood flow (rCBF), diffusion metrics, and dynamic time–signal intensity curve parameters were extracted. Group comparisons were performed using nonparametric statistical tests. Machine learning models, including linear discriminant analysis (LDA), were developed using patient-level grouped nested cross-validation to avoid data leakage. Diagnostic performance was evaluated using out-of-fold receiver operating characteristic (ROC) analysis, calibration assessment, and clinically oriented thresholds prioritizing metastasis sensitivity. **Results:** rCBV_mean and rCBF_mean were significantly higher in meningiomas than in dural metastases (median rCBV_mean: 4.71 vs. 2.95; median rCBF_mean: 3.44 vs. 2.02; both *p* < 0.001). Diffusion metrics and dynamic perfusion parameters, including wash-in time, percentage signal recovery, and wash-out slope, did not differ significantly between groups (*p* > 0.05). Univariate ROC analysis demonstrated strong discrimination for both rCBF_mean (AUC: 0.82; 95% CI: 0.72, 0.90) and rCBV_mean (AUC: 0.82; 95% CI: 0.72, 0.91). An LDA model integrating rCBF_mean and rCBV_mean achieved an out-of-fold AUC of 0.81 (95% CI: 0.72, 0.89) and improved specificity (85%) at a fixed metastasis sensitivity of 85%. **Conclusions:** DSC perfusion MRI-derived rCBF and rCBV are robust biomarkers for differentiating IDMs from meningiomas. An interpretable machine learning model integrating these parameters improves diagnostic specificity while maintaining high sensitivity.

## 1. Introduction

Differentiating intracranial dural metastases (IDMs) from meningiomas poses a diagnostic challenge in neuroradiology due to their overlapping MRI features, such as dural-based enhancement and vasogenic edema [1,2]. Conventional MRI sometimes lacks the reliability to distinguish between the two as their characteristic features, like calcification in meningiomas and necrosis in metastases, can overlap [3,4,5].

The most frequent dural metastases originate from breast, prostate, and lung carcinomas. Although imaging characteristics may vary according to the primary tumor type, several overlapping features with meningiomas are commonly observed, including intense contrast enhancement related to neoangiogenesis and occasional dural tail appearance. On MR spectroscopy, dural metastases typically demonstrate low N-acetylaspartate (NAA)/creatine ratios and elevated lipid peaks while lacking the alanine peak that is more characteristic of meningiomas. Furthermore, certain metastatic subtypes may exhibit relatively high perfusion patterns, further complicating differentiation [6]. These overlapping imaging features highlight the need for quantitative perfusion-based approaches to improve diagnostic discrimination [7].

Perfusion-weighted MRI offers a potential solution by assessing tumor vascularity. Dynamic susceptibility contrast (DSC) MRI enables quantitative assessment of relative cerebral blood volume (rCBV) and relative cerebral blood flow (rCBF), parameters that have been extensively investigated in dural lesions [3,8]. While some studies have reported higher rCBV values in meningiomas compared to IDMs [9], others have shown a significant overlap in rCBV measurements, particularly in hypervascular metastases [3].

Beyond rCBV and rCBF, additional DSC-derived dynamic parameters, including wash-in and wash-out characteristics, have been explored to improve diagnostic performance. Shorter normalized wash-out times have been reported in IDMs in some studies, whereas others found limited discriminatory value for dynamic perfusion or diffusion parameters such as apparent diffusion coefficient (ADC) [5,10].

Machine learning (ML) approaches provide a suitable framework for modeling complex, nonlinear relationships between quantitative biomarkers. By integrating DSC perfusion parameters within supervised classification models, ML has the potential to improve diagnostic accuracy while preserving clinical interpretability. Accordingly, the aim of this retrospective study is to evaluate the diagnostic value of DSC perfusion MRI parameters and to assess whether a simple ML-based model can enhance differentiation between IDMs and meningiomas in a clinically applicable manner.

## 2. Materials and Methods

This retrospective study was approved by the local institutional review board (2024/541), and the requirement for informed consent was waived due to the retrospective study design, in accordance with the Declaration of Helsinki.

### 2.1. Statistical Analysis

Categorical variables were summarized as frequencies and percentages, and comparisons between groups were conducted using the chi-square test. For continuous variables, means, standard deviations, and 95% CIs were calculated. Continuous variables were assessed for normality using the Shapiro–Wilk test. As most parameters did not follow a normal distribution, continuous data are presented as medians with first and third quartiles (Q1–Q3). A two-tailed *p*-value < 0.05 was considered statistically significant. Cohen’s d was used as the primary effect size measure for pairwise comparisons, and eta-squared (η^2^) was reported as a complementary measure. All statistical analyses and visualizations were performed using Python (version 3.11.5; Python Software Foundation, Wilmington, DE, USA), with the assistance of the scipy, statsmodels, pandas, seaborn, and matplotlib libraries.

### 2.2. Patient Selection and Diagnostic Criteria

In this retrospective study, the institutional radiology database was retrospectively searched for patients who underwent brain MRI between November 2015 and September 2024 for evaluation of dural-based intracranial lesions. Patients with IDM or meningioma were classified using histopathological confirmation and clinical–radiological criteria. Histopathological diagnosis served as the reference when available. IDM was diagnosed without histopathological confirmation if there was a known systemic primary malignancy, the dural lesion was absent on prior MRIs, and there was interval development with progressive lesion growth consistent with metastatic disease. Meningioma was diagnosed in the absence of histopathological confirmation when no primary malignancy existed, the dural lesion exhibited typical imaging features, and stability in size and appearance was confirmed through follow-up MRIs. Patients were excluded if they had incomplete MRI exams, significant motion artifacts, or lesions with extensive hemorrhage or necrosis that hindered obtaining reliable measurements. The study involved 56 patients and 65 lesions: 18 with IDM (27 lesions) and 38 with meningiomas (single lesion in each). Histopathological confirmation was not available for all lesions because surgical intervention or biopsy is not routinely performed for every dural-based lesion in clinical practice, particularly in patients with known systemic malignancy or lesions with characteristic imaging features and stable follow-up findings. In such cases, diagnosis was established based on multidisciplinary clinical–radiological consensus and imaging follow-up. This approach reflects real-world diagnostic decision-making in neuro-oncology practice.

### 2.3. MRI Acquisition Protocol

All MRI examinations were performed using either a 1.5 T MRI scanner (Aera, Siemens Healthcare, Erlangen, Germany) equipped with an 18-channel head coil or a 3-T MRI scanner (Skyra, Siemens Healthcare, Erlangen, Germany) equipped with a 32-channel head coil. The routine brain tumor MRI protocol included non-enhanced conventional cranial MRI sequences, DSC perfusion imaging, and contrast-enhanced T1-weighted axial and coronal images following intravenous administration of a gadolinium-based contrast agent (0.1 mmol/kg). The contrast agent was injected at a rate of 3–5 mL/s, followed by a saline flush. DSC perfusion imaging was performed using a T2-weighted gradient-echo echo-planar imaging sequence*. A preload contrast dose was not administered as preload administration may influence contrast leakage-related parameters, particularly percentage signal recovery (PSR).

### 2.4. Image Evaluation and Perfusion Analysis

All MRI examinations were independently reviewed and subsequently evaluated by consensus between two neuroradiologists with 12 and 5 years of experience in neuroradiology. Perfusion data were transferred to a dedicated workstation running commercially available software for DSC perfusion imaging analysis (syngo.via VB30, Siemens Healthcare, Erlangen, Germany). Following evaluation of conventional MRI sequences, post-processing of DSC perfusion imaging data was performed. Time–signal intensity curves were generated for each lesion. An arterial input function was manually selected from the middle cerebral artery, and CBV and CBF maps were generated. Regions of interest (ROIs) were placed within the enhancing solid portion of each lesion, avoiding necrotic or hemorrhagic areas, with reference ROIs in contralateral normal-appearing white matter. From these ROIs, mean CBV and CBF values were recorded. Relative perfusion parameters (rCBV and rCBF) were calculated by normalizing lesion values to corresponding WM values.

Time–signal intensity curves were generated for both lesion and WM ROIs. Wash-in time was defined as the time interval from baseline to the point of maximal signal drop. Relative wash-in time (rWiT) was calculated as the ratio of lesion wash-in time to WM wash-in time.

PSR was calculated using the classical formula:

PSR = (S1 − Smin)/(S0 − Smin)

where S0 represents the baseline signal intensity before contrast administration, S min is the minimum signal intensity at peak contrast-induced signal drop, and S1 is the signal intensity measured at a predefined late time point (90 s after contrast injection). Relative PSR (rPSR) was obtained by normalizing lesion PSR values to those measured in the contralateral normal-appearing white matter.

The wash-outslope was defined as a quantitative measure of the rate of signal recovery following the first-pass contrast bolus and was calculated as:

Wash-out slope = (S1 − Smin)/(t1 − tmin)

where tmin corresponds to the time point at which the minimum signal intensity (Smin) occurs, and t1 represents the time point at which the late signal intensity (S1) was measured. This parameter reflects the speed of post-bolus signal recovery and provides complementary information to PSR regarding contrast leakage and vascular permeability characteristics of the lesion.

For ADC measurements, regions of interest (ROIs) were manually defined in the solid contrast-enhanced portion of each lesion on the corresponding ADC maps. ROI size was adjusted to lesion dimensions to include as representative solid tumor tissue as possible while carefully avoiding cystic, necrotic, hemorrhagic, and calcified areas. Additionally, a subgroup analysis was conducted to compare breast cancer metastases in the IDM cohort with metastases originating from other primary tumors.

### 2.5. Machine Learning Models (LDA, L2 Logistic Regression, Linear SVM)

ML analysis were performed to differentiate IDMs from meningiomas using parameters derived from perfusion MRI. Three models were assessed: Linear Discriminant Analysis (LDA), L2-regularized Logistic Regression, and Linear Support Vector Machine (Linear SVM). Patient-level grouped cross-validation was employed to prevent data leakage, with nested cross-validation performed for model training and evaluation. Evaluation utilized a 5-fold GroupKFold method for unbiased performance estimation and hyperparameter tuning, which was conducted through Optuna. The primary metric for evaluation was the area under the receiver operating characteristic curve (ROC), with models incorporating median imputation and feature standardization. Performance assessment was limited to outer test folds, resulting in ROC curves for performance stability analysis. Two feature sets were evaluated to understand the impact of model complexity on diagnostic performance: a reduced feature set with two parameters (rCBF_mean and rCBV_mean) selected for their statistical significance and clinical relevance, and an extended feature set with seven parameters (including ADC, wash-in time, PSR, wash-out slope, and gender) providing a more comprehensive analysis of perfusion, diffusion, and demographic factors. All three machine learning models were trained and evaluated separately using both feature sets under identical nested cross-validation and hyperparameter optimization procedures. Univariate analysis of perfusion biomarkers rCBF_mean and rCBV_mean was conducted alongside ML-based classification. ROC curve analysis, performed separately for each biomarker using lesion-level data, calculated the area under the curve (AUC) to assess their ability to distinguish between IDMs and meningiomas. Out-of-fold (OOF) predicted probabilities from the outer folds were aggregated to construct overall ROC curves and to support clinically oriented decision threshold analyses. To adjust for within-patient correlations from multiple lesions, cluster-based bootstrap resampling was used to derive 95% confidence intervals for the AUCs. A clinically relevant threshold was chosen to ensure a minimum sensitivity of 85% for metastasis detection, with corresponding sensitivity and specificity values reported for each threshold. Because some patients with IDM had multiple lesions, the primary unit of inference in this study was lesion-level classification. To avoid data leakage, patient-level grouped cross-validation (GroupKFold) was applied, ensuring that lesions from the same patient were not simultaneously included in both training and testing sets. For univariate ROC analyses performed at the lesion level, cluster-based bootstrap resampling at the patient level was used to account for within-patient dependence and to obtain robust confidence intervals. Linear models were deliberately preferred to ensure interpretability and reduce the risk of overfitting given the relatively small sample size. More complex non-linear approaches, such as tree-based or ensemble methods, were not explored to maintain model transparency and clinical interpretability.

As a sensitivity analysis addressing, additional validation strategies were performed using the same two-feature set (rCBF_mean and rCBV_mean). Specifically, we implemented (i) grouped 10-fold nested cross-validation and (ii) leave-one-patient-out cross-validation (LOPO), ensuring that lesions from the same patient were never split across training and test sets. Hyperparameter tuning was conducted within inner grouped cross-validation loops in all settings to prevent data leakage.

In addition to the initially tested linear models (LDA, L2-regularized logistic regression, and linear SVM), we evaluated a decision tree classifier and a k-nearest neighbors (KNN) model. Furthermore, a soft-voting ensemble combining the two best-performing base models (based on out-of-fold AUC) was implemented using averaged predicted probabilities. Model performance was assessed using pooled out-of-fold ROC–AUC, Brier score, and threshold-based clinical metrics. Machine learning analyses were implemented using Python (version 3.11.5) with scikit-learn and Optuna libraries.

## 3. Results

### 3.1. Patient and Lesion Characteristics

A total of 56 patients (mean age: 57.6 ± 11.2 years; 20 men) were included in the study. Among them, 18 patients had IDM, contributing a total of 27 IDM lesions, whereas 38 patients had meningiomas, with one lesion per patient, resulting in 38 meningioma lesions. Overall, 65 lesions were analyzed.

Among the IDMs, the most common primary tumor origin was breast cancer (12 lesions, 44.4%), followed by GBM (7 lesions, 25.9%), colon cancer (3 lesions, 11.1%), lung cancer (2 lesions, 7.4%), and one lesion each from medulloblastoma, melanoma, and KML (3.7% each). The cohort consisted of 36 females (64.2%) and 20 males (35.8%) based on patient count.

### 3.2. Perfusion and Diffusion Parameters in IDMs and Meningiomas

**Figure 1:**
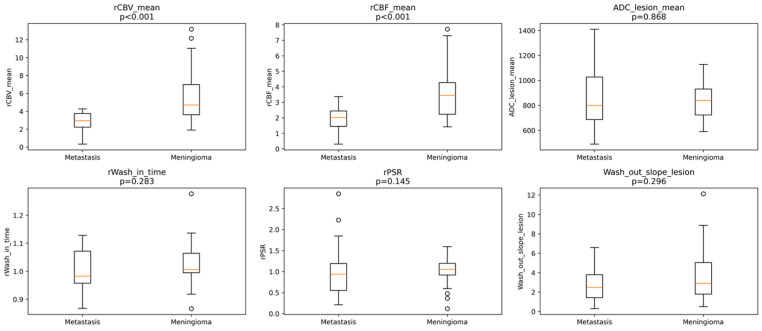
Perfusion, diffusion, and dynamic DSC parameters in dural metastases and meningiomas. Boxplots show the distribution of DSC-derived perfusion, diffusion, and dynamic parameters in dural metastases and meningiomas. rCBV_mean and rCBF_mean were significantly higher in meningiomas (*p* < 0.001), whereas ADC_lesion_mean, relative wash-in time (rWiT), relative percentage signal recovery (rPSR), and wash-out slope did not differ significantly between groups. The central orange line indicates the median value, and the circles represent outliers.

Relative perfusion parameters differed significantly between IDMs and meningiomas. Median rCBV_mean was significantly higher in meningiomas compared with IDMs (4.71 [IQR 3.71] vs. 2.95 [IQR 1.54], *p* < 0.001) (Figure 1).

**Figure 2:**
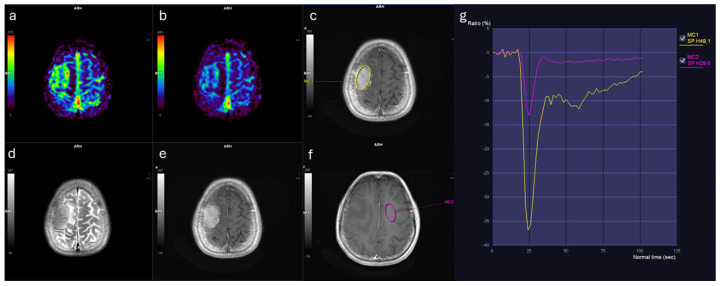
Brain MRI findings in a female patient with solitary IDM from breast cancer. (**a**) Relative cerebral blood volume (rCBV) map shows mildly increased perfusion. (**b**) Relative cerebral blood flow (rCBF) map demonstrates corresponding flow increase. (**c**) Contrast-enhanced T1-weighted image with region of interest (ROI, yellow contour) placed within the enhancing lesion. (**d**) T2-weighted image reveals lesion morphology. (**e**) Contrast-enhanced T1-weighted image illustrates enhancement pattern. (**f**) Contrast-enhanced T1-weighted image with ROI in contralateral normal-appearing white matter (pink contour). (**g**) Time–signal intensity curves: lesion (yellow curve) shows altered contrast dynamics compared to white matter (pink curve).

**Figure 3:**
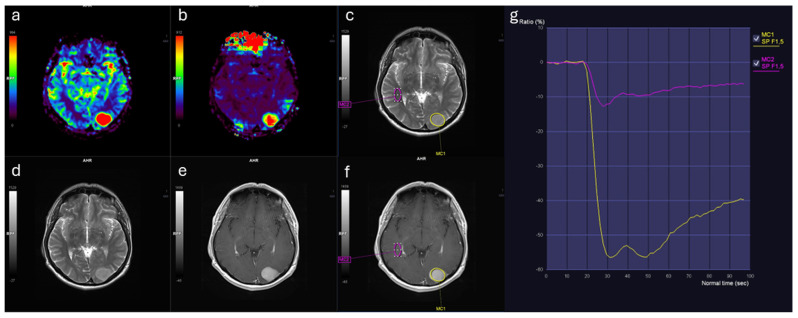
Brain MRI findings in a male patient with atypical meningioma (WHO Grade 2). (**a**) Relative cerebral blood volume (rCBV) map demonstrates markedly increased perfusion. (**b**) Relative cerebral blood flow (rCBF) map shows corresponding elevated flow. (**c**) T2-weighted image with region of interest (ROI, yellow contour) placed within the solid portion of the lesion. (**d**) T2-weighted image illustrates lesion morphology. (**e**) Contrast-enhanced T1-weighted image depicts homogeneous enhancement. (**f**) Contrast-enhanced T1-weighted image with ROI in contralateral normal-appearing white matter (pink contour). (**g**) Time–signal intensity curves: lesion (yellow curve) reveals higher perfusion and distinct kinetics compared to white matter (pink curve).

Similarly, rCBF_mean values were markedly elevated in meningiomas (3.44 [IQR: 2.11]) relative to IDMs (2.02 [IQR: 1.10], *p* < 0.001) (Table 1). Both rCBF_mean and rCBV_mean demonstrated large effect sizes (Cohen’s d > 1.1; η^2^ ≈ 0.35), indicating marked separation between groups. In contrast, no significant differences were observed between groups in terms of ADC_lesion_mean, rWiT, rPSR, or wash-out slope (Figure 1). Representative perfusion maps and time–signal intensity curves for IDM and meningioma lesions are shown in Figure 2 and Figure 3.

### 3.3. Subgroup Analysis of IDM According to Primary Tumor Origin

A subgroup analysis was performed within the IDM cohort to explore potential differences between breast cancer metastases (n = 12) and metastases from other primary tumors (n = 15). Within the IDM cohort, a subgroup analysis was performed to investigate potential differences between breast cancer metastases (n = 12) and metastases from other primary tumors (n = 15). No statistically significant differences were observed between these subgroups in rCBV_mean (*p* = 0.064), rCBF_mean (*p* = 0.407), ADC_lesion_mean (*p* = 0.380), rWiT (*p* = 0.241), rPSR (*p* = 0.242), or wash-out slope (*p* = 0.223).

### 3.4. Diagnostic Performance of Perfusion Biomarkers and Machine Learning Models

**Figure 4:**
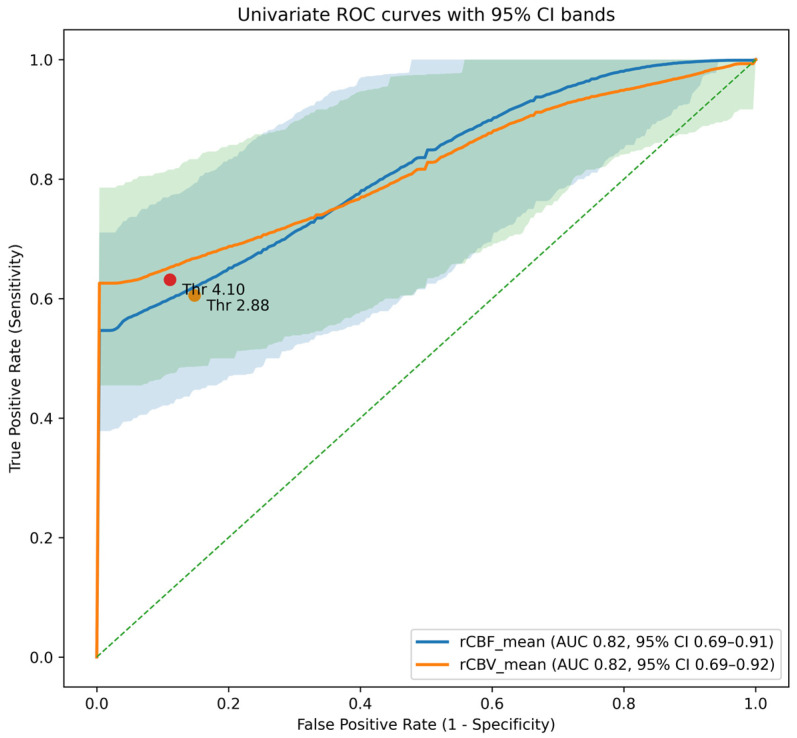
Receiver operating characteristic (ROC) curves for univariate perfusion parameters (rCBF_mean and rCBV_mean) in differentiating intracranial dural metastases from meningiomas. The blue and orange solid lines represent ROC curves for rCBF_mean and rCBV_mean, respectively. The corresponding shaded areas indicate their 95% confidence intervals derived using patient-level cluster bootstrap resampling. Markers indicate clinically oriented threshold values selected to prioritize metastasis sensitivity (rCBF_mean < 2.88 and rCBV_mean < 4.10). The diagonal dashed line represents the line of no discrimination. These findings suggest that perfusion parameters, particularly rCBF and rCBV, may be clinically useful non-invasive biomarkers for differentiating dural metastases from meningiomas.

Univariate ROC analysis revealed statistically significant differences in both rCBF_mean and rCBV_mean between IDMs and meningiomas. The AUC for rCBF_mean was 0.82 (95% CI: 0.72–0.90) and 0.82 for rCBV_mean (95% CI: 0.73–0.91) (Figure 4). Using clinically oriented thresholds prioritizing metastasis detection (minimum sensitivity of 85%), rCBF_mean < 2.88 and rCBV_mean < 4.1 were identified as optimal cut-off values. These thresholds yielded metastasis sensitivities of 85%, with corresponding meningioma specificities of 61% and 66%, respectively.

**Figure 5:**
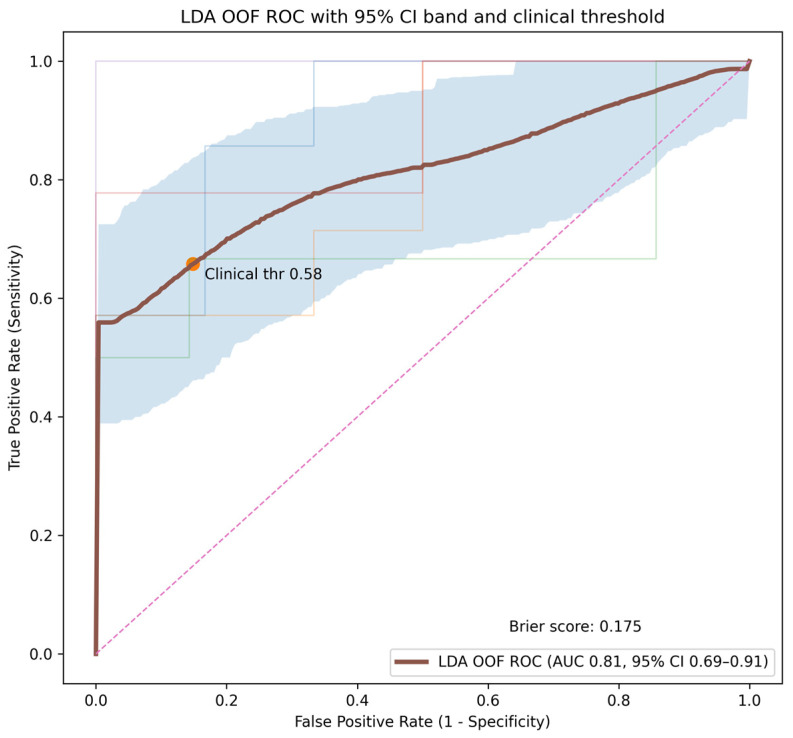
Receiver operating characteristic (ROC) curve of the linear discriminant analysis (LDA) model based on nested cross-validation. The bold curve represents the pooled out-of-fold ROC performance. The bold dark curve represents the pooled out-of-fold ROC performance. The light blue shaded region indicates the 95% confidence interval derived using patient-level cluster bootstrap resampling. The faint colored curves represent ROC performance across individual outer cross-validation folds. The marker indicates the clinically oriented decision threshold (0.58), and the Brier score reflects overall probabilistic calibration performance. The diagonal dashed line represents the line of no discrimination. The model demonstrates clinically meaningful discrimination, supporting the potential role of machine learning-based perfusion analysis in non-invasive lesion characterization.

When using a two-parameter feature set, LDA exhibited the best diagnostic performance, with an average fold-level AUC of approximately 0.85 and an OOF-AUC of 0.81, resulting in a Brier score of 0.17 (Figure 5). Using a clinically oriented decision threshold to maintain metastasis sensitivity at approximately 85%, the 2-feature LDA model achieved a meningioma sensitivity of 68.4% with an accuracy of 75.3%, outperforming the individual perfusion parameters. Linear SVM and L2-regularized logistic regression demonstrated comparable but slightly lower performances (Table 2).

## 4. Discussion

Meningioma is the most common meningeal tumor, often detected incidentally on MRI. Most are asymptomatic when small; however, larger tumors can cause neurological symptoms like headaches [5]. On a standard MRI, a typical metastasis and a typical meningioma appear different from a radiological standpoint. They appear as spherical or plaque-like formations extending along the dura mater, typically separated from brain parenchyma by a cerebrospinal fluid cleft. Characteristically, meningiomas exhibit intense and uniform contrast enhancement in 60–72% of cases, often associated with dural thickening known as the “dural tail” [11]. Calcifications occur in approximately 25% of cases [12], indicating slow growth and low malignancy [13], while 20% of patients show focal reactive hyperostosis [12,14]. Meningiomas can invade the dural sinus and adjacent bones, with nearly 50% accompanied by peritumoral edema [14]. They demonstrate high signal on DWI with low ADC values, suggesting high cellular density, though malignancy may lower ADC values further [15].

IDMs present with variable perivascular vasogenic edema and localized nodular dural thickening, often causing compression of brain parenchyma [15]. Post-contrast imaging reveals intense paramagnetic material accumulation, with hemorrhages and the “dural tail” phenomenon occurring in about half of cases. Enhanced dispersion is more common in IDMs [9,16,17], and minimal ADC values indicate enhanced cellularity, but their correlation with histological types of metastasis remains debatable [18]. Metastases can exhibit either hypo- or hypervascular perfusion, largely hypervascular in cases from renal carcinoma, melanoma, and neuroendocrine carcinoma [19]. Distinguishing meningiomas from other conditions, such as IDMs, remains challenging, despite imaging advancements, complicating treatment decisions [19,20,21]. Approximately 2% of surgically removed dural masses initially diagnosed as meningiomas are actually mimic diseases, mostly metastases [11]. The incidence of IDM is difficult to determine, as it occurs in 8–9% of patients with extraneural malignancies and while about 20% of IDM cases are asymptomatic [16]. IDMs are responsive to systemic chemotherapy because they are located outside the blood–brain barrier in contrast to parenchymal and leptomeningeal metastases [22]. As a result, preoperative distinction is required to choose the course of treatment in these situations

Our study evaluated the ability of DSC perfusion parameters and ML models to differentiate between IDMs and meningiomas. The principal findings indicate that rCBF and rCBV serve as effective discriminators, with meningiomas showing notably higher values. While these univariate biomarkers alone offer good diagnostic accuracy (AUC ~0.82), a multivariate LDA model that combines both parameters delivered improved specificity with clinically relevant sensitivity. Other measures, like ADC and various dynamic contrast-enhanced metrics (rWiT, PSR, wash-out slope), did not provide significant additional discrimination in our sample. These findings highlight the complexity of perfusion-based differentiation and the potential for machine learning to translate this knowledge into a clinically useful tool.

Previous studies investigating DSC perfusion in dural lesions have reported heterogeneous findings, with some demonstrating higher rCBV values in meningiomas compared with dural metastases, while others reported overlapping perfusion characteristics between tumor types [8,23,24]. Overall, the literature suggests that perfusion metrics are influenced by tumor vascularity, histologic composition, and imaging methodology, which may contribute to variability across studies.

The higher rCBV and rCBF values observed in meningiomas can be explained by differences in tumor vascular architecture. Meningiomas typically demonstrate dense capillary networks and prominent neoangiogenesis with relatively organized vascular channels, resulting in increased microvascular density and perfusion [8,25]. In contrast, dural metastases often show more heterogeneous vascularity depending on the primary tumor origin and may exhibit less organized or more variable vascular structures, which can lead to lower or more variable perfusion measurements [26,27]. These differences in vascular microenvironment likely explain the consistent elevation of rCBV and rCBF in meningiomas observed in our cohort.

Our findings differ from those reported by Wu et al., who observed higher CBV and CBF values in solitary dural metastases compared with meningiomas [5]. Several factors may explain this discrepancy, including differences in DSC acquisition protocols, normalization methods, tumor composition, and the distribution of metastatic primary tumor types. Hypervascular metastases, such as those originating from melanoma or renal cell carcinoma, may substantially influence perfusion measurements and contribute to divergent results across studies. Additionally, methodological differences in ROI placement and cohort characteristics may further explain these inconsistencies.

Our study has several strengths, including the use of patient-level grouped nested cross-validation to prevent data leakage and provide robust performance estimates, rigorous bootstrap confidence intervals for univariate AUCs, and a clinically oriented evaluation using sensitivity-prioritized thresholds. However, limitations must be acknowledged. First, the retrospective single-center design and relatively small sample size, particularly within the metastasis subgroup, may limit the generalizability of the findings and the stability of machine learning performance estimates. Although nested cross-validation was applied to reduce overfitting, external validation in larger multicenter cohorts is required. Second, not all lesions were histopathologically confirmed. Reliance on clinical–radiological diagnosis in a subset of cases may introduce potential misclassification bias; however, this reflects routine clinical practice and enabled inclusion of a representative cohort of dural-based lesions. Third, the use of both 1.5 T and 3 T MRI scanners may introduce variability in perfusion measurements. Although normalization to contralateral normal-appearing white matter was performed to reduce inter-scanner variability, residual differences related to field strength cannot be excluded. Future studies should focus on prospective multicenter validation with standardized imaging protocols, systematic histopathological confirmation, and stratification according to primary tumor type and meningioma histological subtype. Integration of additional imaging approaches, such as arterial spin labeling or radiomic features derived from perfusion and conventional sequences, may further improve diagnostic performance.

## 5. Conclusions

In conclusion, our study supports the role of DSC perfusion MRI, specifically rCBF and rCBV, as valuable biomarkers for differentiating IDMs from meningiomas, with meningiomas showing higher perfusion in our cohort. The application of a simple linear ML model effectively synthesized this information, achieving an excellent balance of sensitivity and specificity that surpasses the use of either parameter alone. Despite ongoing contradictions in the literature, which likely reflect underlying pathological and technical diversity, a focused, clinically guided analysis of perfusion data can provide significant diagnostic leverage. Integrating such models into the neuroradiological workflow has the potential to improve preoperative diagnostic confidence, thereby guiding more appropriate patient management.

## Figures and Tables

**Table 1:** Comparison of perfusion, diffusion, and dynamic MRI parameters between dural metastases and meningiomas.

Parameter	Dural Metastases (n = 27) Median (Q1–Q3)	Meningiomas (n = 38) Median (Q1–Q3)	Mann–Whitney U	*p* Value
rCBV_mean	2.95 (2.22–3.75)	4.71 (3.61–6.99)	188	<0.001
rCBF_mean	2.02 (1.44–2.44)	3.44 (2.23–4.27)	184	<0.001
ADC_lesion_mean	800 (687–1027)	840 (723–931)	500	0.868
rWash_in_time	0.98 (0.96–1.07)	1.01 (0.99–1.06)	432	0.283
rPSR	0.94 (0.55–1.19)	1.05 (0.92–1.20)	403	0.145
Wash_out_slope_lesion	2.48 (1.43–3.79)	2.89 (1.79–5.05)	434	0.296

rCBV: relative Cerebral Blood Volume, rCBF: relative Cerebral Blood Flow, ADC: Apparent Diffusion Coefficient, rPSR: relative Percentage Signal Recovery.

**Table 2:** Diagnostic performance of individual perfusion parameters and machine learning models.

Method	Threshold	ROC-AUC	Metastasis Sensitivity (Class 0)	Meningioma Sensitivity (Class 1)	Accuracy	Balanced Accuracy
rCBF_mean	2.88	0.82	85.2%	60.5%	70.8%	n/a
rCBV_mean	4.10	0.82	88.9%	63.2%	73.8%	n/a
LDA (2-feature)	0.58	0.81	85.2%	68.4%	75.3%	76.8%
Linear SVM (2-feature)	0.66	0.80	85.2%	63.2%	72.3%	74.2%
Logistic Regression L2 (2-feature)	0.68	0.80	85.2%	65.8%	73.8%	75.5%

rCBF: relative Cerebral Blood Flow, rCBV: relative Cerebral Blood Volume, LDA: Linear Discriminant Analysis, AUC: Area Under the Curve. Threshold-based results are reported using class-specific sensitivity for metastasis (class 0) and meningioma (class 1). Machine learning model thresholds were selected to maintain metastasis sensitivity ≥ 85%. ROC-AUC for rCBF_mean and rCBV_mean is based on lesion-level univariate ROC; ROC-AUC for ML models is based on pooled out-of-fold predictions.

## Data Availability

The data presented in this study are available on request from the corresponding author. The data are not publicly available due to privacy and ethical restrictions.

## Abbreviations

The following abbreviations are used in this manuscript:

IDMs	Intracranial Dural Metastases
DSC	Dynamic Susceptibility Contrast
rCBV	relative Cerebral Blood Volume
rCBF	relative Cerebral Blood Flow
ADC	Apparent Diffusion Coefficient
ML	Machine Learning
PSR	Percentage Signal Recovery
ROIs	Regions of Interest
rWiT	relative Wash-in Time
LDA	Linear Discriminant Analysis
SVM	Support Vector Machine
ROC	Receiver Operating Characteristic Curve
AUC	Area Under the Curve
OOF	Out-of-Fold
